# Cytomegalovirus viraemia is associated with poor growth and T-cell activation with an increased burden in HIV-exposed uninfected infants

**DOI:** 10.1097/QAD.0000000000001568

**Published:** 2017-07-27

**Authors:** Miguel A. Garcia-Knight, Eunice Nduati, Amin S. Hassan, Irene Nkumama, Timothy J. Etyang, Naseem J. Hajj, Faith Gambo, Denis Odera, James A. Berkley, Sarah L. Rowland-Jones, Britta Urban

**Affiliations:** a Centro de Investigaciones en Ciencias Microbiologicas, Benemerita Universidad Autonoma de Puebla, Cuidad Universitaria, Puebla, Mexico; b KEMRI-Wellcome Trust Research Program, Centre for Geographical Medicine Research; c Comprehensive Care and Research Clinic, Kilifi County Hospital, Kilifi, Kenya; d NDM Research Building, University of Oxford, Oxford; e Liverpool School of Tropical Medicine, Liverpool, UK.

**Keywords:** anthropometry, cytomegalovirus, HIV exposed-uninfected, HIV-1, infant, T cell

## Abstract

**Objective:**

Factors associated with poor health in HIV-exposed-uninfected (HEU) infants are poorly defined. We describe the prevalence and correlates of cytomegalovirus (CMV) viraemia in HEU and HIV-unexposed-uninfected (HUU) infants, and quantify associations with anthropometric, haematological, and immunological outcomes.

**Design:**

Cross-sectional, including HEU and HUU infants from rural coastal Kenya.

**Methods:**

Infants aged 2–8 months were studied. The primary outcome was CMV viraemia and viral load, determined by quantitative PCR. Correlates were tested by logistic and linear regression; coefficients were used to describe associations between CMV viraemia and clinical/immunological parameters.

**Results:**

In total, 42 of 65 (64.6%) infants had CMV viraemia [median viral load, 3.0 (interquartile ranges: 2.7–3.5) log_10_ IU/ml]. Compared to community controls, HEU infants had six-fold increased odds of being viraemic (adjusted odds ratio 5.95 [95% confidence interval: 1.82–19.36], *P* = 0.003). Age, but not HEU/HUU status, was a strong correlate of CMV viral load (coefficient = −0.15, *P* = 0.009). CMV viral load associated negatively with weight-for-age (WAZ) Z-score (coefficient =  −1.06, *P* = 0.008) and head circumference-for-age Z-score (coefficient =  −1.47, *P* = 0.012) and positively with CD8^+^ T-cell coexpression of CD38/human leucocyte antigen DR (coefficient = 15.05, *P* = 0.003).

**Conclusion:**

The odds of having CMV viraemia was six-fold greater in HEU than HUU infants when adjusted for age. CMV viral load was associated with adverse growth and heightened CD8^+^ T-cell immune activation. Longitudinal assessments of the clinical effects of primary CMV infection and associated immunomodulation in early life in HEU and HUU populations are warranted.

## Introduction

Successful prevention of mother-to-child transmission (PMTCT) of HIV-1 strategies have given rise to an increased number of HIV-exposed uninfected (HEU) infants over the past decade [1]. Despite these gains, higher morbidity and mortality is observed in HEU infants compared with infants born to HIV-uninfected mothers (referred to as HIV-unexposed uninfected [HUU]) [2–5]. Defining the mechanisms of poor health in this population is an increasing public health priority.

Numerous factors may disproportionately impact health outcomes in HEU infants, including heightened susceptibility to infections [6–8] (which are associated with maternal immunopathology [6]), exposure to antiretroviral therapy (ART) and exposure to a maternal immune system perturbed by HIV-1 infection (reviewed in [9]). A relatively unexplored influence is exposure to maternal coinfections. Cytomegalovirus (CMV) is a common maternal coinfection with high seroprevalence, especially in countries that also have high HIV-1 prevalence (80–100% in pregnant women) [10,11].

Most vertical CMV transmission occurs postnatally, through contact with breast milk, maternal saliva, urine, or other bodily fluids. Immunocompetent full-term infants who acquire CMV postnatally benefit from passive maternal antibodies and generate protective immunity leading to what is usually an asymptomatic life-long latent infection. However, in-utero CMV transmission is a leading cause of congenital disease and can lead to birth defects, neurodevelopmental deficiencies, and hearing loss [12]. In addition, primary postnatal infection in immunocompromised hosts, including preterm infants, can lead to a range of clinical manifestations [13]. Infant HIV-1/CMV coinfection has been reported to be particularly severe, with higher viral loads of both infections, accelerated disease progression and increased mortality compared with CMV-uninfected HIV-1-positive infants [14–16]. The effects of primary postnatal CMV infection in HEU infants, a population with immunological alterations, remain poorly understood.

HEU infants exhibit reduced transfer of specific maternal immunoglobulin G antibodies *in utero*[17,18] which may lead to reduced protection from CMV infection and delayed control of viremia. In addition, phenotypic alterations occur in T cells from HEU infants, including expansions of activated and differentiated cells and reductions in circulating naïve cells, particularly among CD8^+^ cells [19,20]. Strikingly, CMV infection has broad effects on the T-cell compartment in heathy Gambian infants [21] and Malawian children [22], with increased expression of differentiation, activation, and senescence markers that are associated with functional impairments and reduced vaccine responses in elderly adults [23]. We previously observed that ex-vivo CD8^+^ T-cell activation levels varied widely in HEU and HUU Kenyan infants [24]. We, therefore, hypothesized that CMV infection disproportionately affects HEU infants in early life, leading to immunomodulation including enhanced T-cell activation. Consequently, we assessed the prevalence and correlates of peripheral blood CMV viremia, a marker principally of primary CMV infection, in Kenyan HEU and HUU infants from a rural coastal region. In addition, we assess associations between CMV viraemia and anthropometric, haematological, and immunological outcomes including ex-vivo T-cell activation, regulatory T-cell (Treg) frequencies, and frequencies of mature plasmacytoid dendritic cell (pDC).

## Methods

### Study design

A cross-sectional retrospective study design was used. Enrolment specimens were obtained from two groups recruited contemporaneously (from 2011 to 2013) to study immune health in HEU infants [25]. Specimens from the first group were from an observational cohort of HIV-exposed infants attending the Kilifi County Hospital for PMTCT care, a tertiary-level health facility in rural coastal Kenya [26]. PMTCT provision was according to Kenyan national guidelines [27] as previously described [26]. Infants were recruited through a consecutive sampling approach whereby enrolment occurred between 3 and 18 months-of-age, and follow-up occurred at 3-month intervals until 24 months-of-age [25]. The present study was restricted to specimens from HEU infants who were 2–8 months-of-age, who remained HIV-1 negative throughout follow-up and who had a cryopreserved plasma sample (Supplementary Fig. 1). The second group were 28-month-old HUU infants recruited from a secondary-level health facility or during an annual malaria epidemiological survey, both within the Kilifi County Hospital catchment area, that were born to HIV-uninfected mothers and who provided a one-off sample. All infants who met these criteria were included (Supplementary Fig. 1).

HIV-1 infection status among all HIV-exposed infants was determined as per Kenyan national PMTCT guidelines [27]: by a dried blood spot PCR at 6 weeks of age, by rapid antibody test at 9 months and by a confirmatory rapid antibody test at 18 months-of-age. Owing to ethical considerations HUU infants were not directly screened for HIV-1 infection. *N* = 22 infants were born to HIV-negative mothers (tested antenatally). Antenatal screening data was not available for the remaining *N* = 7 infants (all between 6 and 8 months-of-age) classed as HUU. Local HIV-1 prevalence in women aged 25–35 years is 4.1% [28]. The severity of HIV-1 pathology in early infancy and low maternal prevalence relative to the national average in women (7.6% [29]) made recruitment of HIV-1 positive or HEU infants unlikely. Whole blood samples were collected by a clinician at scheduled visits and stored at room temperature until processing. Complete blood counts were determined using a Coulter MDII-18 counter (Beckman-Coulter, Fullerton, California, USA).

### Plasma storage and cytomegalovirus viral load determination

Sample processing was done within 4 h of venepuncture, and plasma was stored continuously at −80°C until use. Viral nucleic acids were extracted from 200 μl of plasma using the QIAamp MiniElute Virus Spin Kit (Qiagen, Limburg, Netherlands). CMV viral load was determined using the RealStar CMV PCR Kit 1.0 (Altona Diagnostics, Hamburg, Germany) following the manufacturer's instructions. Real-time quantitative PCR was done using the ABI Prism 7500 SDS instrument (Applied Biosystems, Waltham, Massachusetts, USA). A single 96-well plate run was used for all HEU and HUU samples, a negative control and four standards calibrated against the first WHO International Standard for Human CMV Nucleic Acid Amplification (these served to generate a standard curve, as quality controls, and as a positive control). The assay limit of detection is estimated to be 92.1 IU/ml. Viraemia was defined as a measurable viral load, as per manufacturer's recommendations, and viral loads are presented as log_10_ IU/ml of peripheral blood.

### Ex-vivo immunophenotyping of leucocytes

Following centrifugation, plasma was removed from packed cells and replaced with supplemented Roswell Park Memorial Institute medium (10% inactivated newborn calf serum, 0.01% β-mercaptoethanol, 1% penicillin/streptomycin, 1% L-glutamate, and 1% HEPES). Fluorescently labelled antibodies in 100 μl were incubated at room temperature. All antibodies were from Becton Dickinson (Franklin Lakes, New Jersey, USA) unless stated: anti-CD3 Pacific Blue, anti-CD3 allophycocyanin (APC)-H7, anti-CD4 Pacific Blue, anti-CD4 peridinin chlorophyll (Biolegend, San Diego, California, USA), anti-CD8 APC-H7, anti-human leucocyte antigen (HLA)-DR fluorescein isothiocyanate (FITC), anti-CD38 phycoerythrin-Cy7 (Biolegend), anti-programmed cell death protein (PD)-1 APC (eBiosciences, San Diego, California, USA), anti CD25 phycoerythrin, forkhead box P3 (FOXP3) FITC (eBiosciences), antilineage FITC, anti-HLA-DR peridinin chlorophyll, anti-CD11c APC, anti-CD123 efluor 450 (eBiosciences), and anti-CD86 phycoerythrin. Red blood cell lysis was done using BD FACS lysing solution (BD Bioscience, Franklin Lakes, New Jersey, USA) and cells were washed once in phosphate buffered saline before acquisition. To detect Tregs the Human Treg Whole Blood staining kit (eBiosciences) was used per the manufacturer's instructions. Following permeabilization, cells were blocked with 2% rat serum and incubated with anti-FOXP3 (clone PCH101). Tregs were defined as CD25^hi^ FOXP3^+^ CD4^+^ T cells (Supplementary Fig. 2D). pDCs were defined as lineage^-^ HLADR^+^ CD123^+^ leucocytes (Supplementary Fig. 3) [30]. Isotype controls (BD Bioscience) matched for concentration and processed under the same conditions as cells were used to set positive gates for all markers except CD3, CD4^+^, CD8^+^, and CD25.

### Flow cytometry

Flow cytometry was done on a Cyan ADP (Beckman Coulter, Brea, California, United States) with three lasers and nine fluorescence channels, with Summit 4.3 software. Single-stain compensation controls were set using antimouse kappa beads (BD Bioscience) and the respective fluorochrome-conjugated antibody for all experiments. A minimum of 70 000 CD3^+^ events and 200 000 events in the leucocyte gate were collected for T cell phenotyping and pDC phenotyping, respectively. To minimize doublet events, event acquisition rate alterations were minimized (Supplementary Fig. 2A).

### Data analysis

Flow cytometry compensation and analysis was done in FlowJo version 7.6 for Windows (Treestar, Ashland, Oregon, USA). FlowJo templates were made for consistent gating. FlowJo tables were exported into STATA v.12 (Stata Corp, College Station, Texas, USA) for analyses.

The 2006 WHO growth standard references [31] were used to generate WAZ, height-for-age (HAZ), weight-for-height and head circumference-for-age SD Z-scores using the STATA igrowup package. A distribution of the participant characteristics was done. Continuous data were presented using medians [interquartile ranges, (IQR)].

The primary outcome of the study is peripheral blood CMV viraemia, and is presented as a prevalence of all infants. Correlates of CMV viremia and CMV viral load were determined using multivariable logistic and linear regression, respectively. Crude and adjusted odds ratio, their 95% confidence interval and likelihood ratio test *P* values are presented. Multivariable linear regression was used to assess the relationships between CMV viral load and anthropometric, haematological immunological outcomes. Whole blood cell counts were log transformed (log_10_) before inclusion in regression models. Multiple comparison correction was made independently for grouped outcomes using the Dunn–Šidák method; because of the limited sample size of the study αwas set at 0.1. For baseline differences, differences in maternal characteristics and in the subset of infants with immunological data, χ^2^ tests were used for categorical variables and unpaired t tests or Mann–Whitney tests were used to compare continuous variables.

### Ethical considerations

The study protocol was reviewed and approved by the Kenyan Medical Research Institute Ethical Research Council (protocol no. SSC 2085) and the Oxford Tropical Research Ethics Committee (approval reference 45–11). Informed consent was obtained from caregivers that accompanied the infants to the health facilities.

## Results

### Study participants

Overall, 65 infants were studied (Table 1): 36 HEU (median age 4.2 months [IQR 3.3 to 4.9]) and 29 HUU (median age 3.5 months [IQR 2.8 to 5.6]). No significant baseline differences between HEU and HUU infants were observed with regards to age and sex, though HEU infants had increased lymphocyte counts (*P* = 0.04).

### HIV exposure is strongly associated with having cytomegalovirus viraemia

A total of 42 of 65 infants were found to be CMV viraemic: 29 of 36 (81%) among HEU and 13 of 29 (45%) among HUU infants (χ^2^ test *P* = 0.004). In multivariate analyses and after adjusting for age and sex, HEU infants had a six-fold increased odds of being CMV viraemic, compared with HUU infants (adjusted odds ratio 5.95 [95% confidence interval: 1.82 to 19.36], *P* = 0.003; Table 2).

### Cytomegalovirus viral load is strongly associated with infant age

**Fig. 1:**
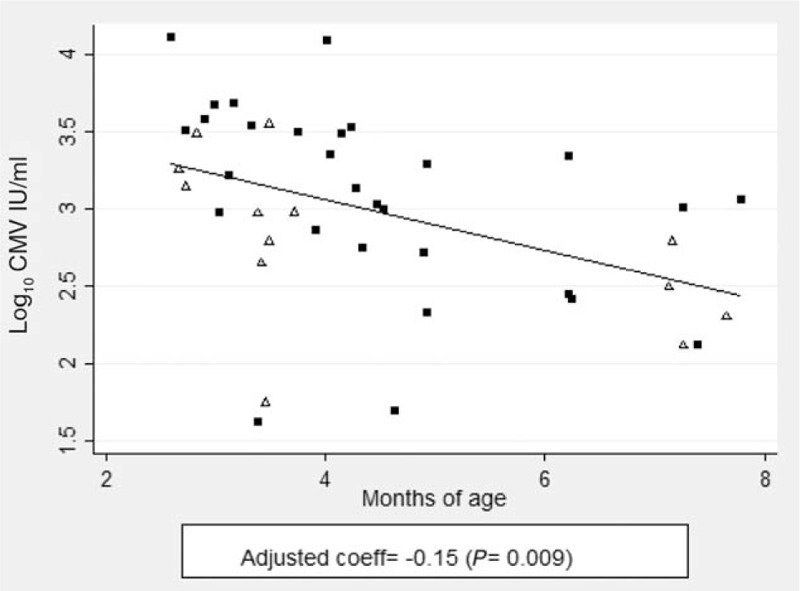
Age is strong correlate for CMV viraemia.

Median viral load among all 42 CMV viraemic infants was 3.0 (IQR: 2.6–3.5) log_10_ IU/ml: 3.1 [IQR: 2.7–3.5] log_10_ IU/ml among HEU infants and 2.7 (IQR: 2.4–3.2) log_10_ IU/ml among HUU infants (*P* = 0.161). HIV exposure status was not associated with CMV viral load (*P* = 0.142) when adjusted for age and sex. Age had a strong negative correlation (coefficient = −0.15 per month of age, *P* = 0.009) with CMV viral load, but there was no association with sex (*P* = 0.55; Table 2 and Fig. 1).

Scatter plot with a linear regression line indicating the relationship between infant age and CMV viral load among 42 CMV viraemic infants. The correlation coefficient is shown in a multivariable regression model following adjustments for HIV exposure and gender. Open triangles represent HIV-unexposed uninfected infants and filled squares HIV-exposed uninfected infants. CMV, cytomegalovirus.

### Anthropometry parameters correlate with cytomegalovirus viraemia

**Fig. 2:**
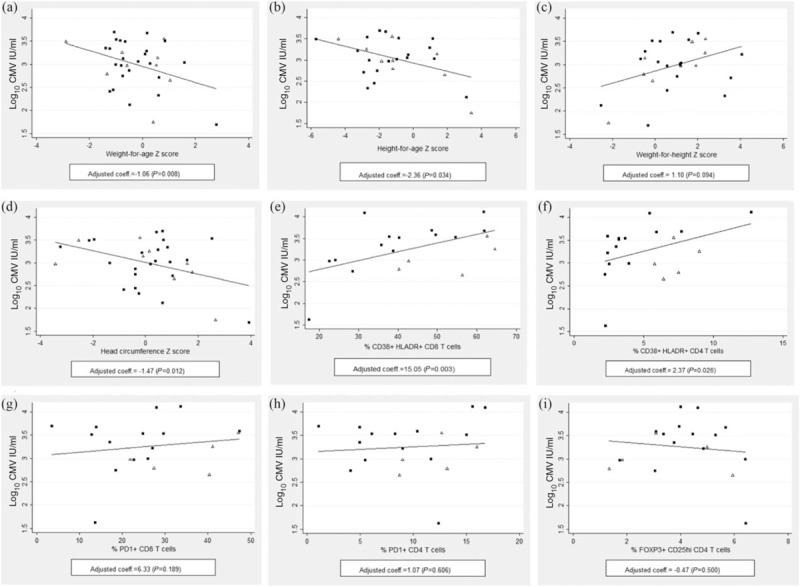
Relationship between CMV viraemia and anthropometry outcomes and T-cell phenotypes.

Having CMV viraemia was not associated with any anthropometry outcome when adjusted for age, sex, HIV exposure, and multiple comparisons (Supplementary Table 1). However, among CMV viraemic infants, CMV viral load had strong effects on WAZ (coefficient = −1.06, *P* = 0.008), head circumference-for-age Z-score (coefficient = −1.47, *P* = 0.012) and a trend toward an effect on HAZ (coefficient = −2.36, *P* = 0.034) following adjustments for age, sex, HIV exposure and multiple comparisons (Table 3 and Fig. 2a, b, and c; significance considered at *P* < 0.025). No effect was observed on weight-for-height following adjustments (coefficient = 1.10, *P* = 0.094; Table 3 and Fig. 2d).

(a–d) Scatter plots with a linear regression line indicating the relationship between CMV viral load and infant weight-for-age, head circumference-for-age, height-for-age and weight-for-height Z-scores (*N* = 33). Following the Dunn–Šidák correction for multiple comparisons, significance was considered at *P* < 0.025. (e–i) Scatter plots with a linear regression line showing for the relationship between CMV viral load and T-cell phenotypes (*N* = 19). Following the Dunn–Šidák correction for multiple comparisons, significance was considered at *P* < 0.017. For all plots, the correlation coefficient is shown in a multivariable regression model following adjustments for age, gender and HIV exposure. For all plots, HUU infants are indicated as open triangles and HEU infants as filled squares. CMV, cytomegalovirus.

### Associations between whole blood cell counts and cytomegalovirus viraemia

A strong positive correlation was seen between having CMV viraemia and lymphocyte percentage (coefficient = 12.08, *P* = 0.007) and a trend toward a negative correlation was seen between having CMV viraemia and neutrophil count (coefficient = −0.29, *P* = 0.033) when adjusted for age, sex, HIV exposure, and for multiple comparisons (Supplementary Table 1; significance considered at *P* < 0.013). No effect was observed in relation to the remaining haematological parameters analysed. No significant effect of CMV viral load was observed on any haematological parameters following adjustments (Table 3).

### Cytomegalovirus viraemia is strongly associated with increased immune activation in ex-vivo CD8^+^ T cells

HLADR/CD38 coexpression on T cells is a useful activation marker in the context of constitutive CD38 expression on infant T cells [32–34]. We found no significant differences between *ex vivo* activated CD8^+^ T cells in 2 to 5-month-old HEU and HUU infants (Table 1); however, we observed wide variation (range: 2.5–75.4%) in the percentage of activated CD8^+^ T cells, irrespective of HIV exposure status. We, therefore, assessed the relationship between CMV viraemia and the frequency of ex-vivo leucocytes, including activated (HLADR^+^ CD38^+^) and PD-1^+^ CD4^+^ and CD8^+^ T cells, Tregs, and mature (CD86^+^) pDCs. This analysis was restricted to the 2 to 5-month-old infants for whom immunophenotypic data had been previously generated using fresh whole blood (Supplementary Fig. 1). These infants did not differ significantly in HIV exposure, sex distribution, growth Z-scores, or haematology from the overall infant population (Supplementary Table 2).

No significant correlation was seen between having CMV viraemia and T cell activation, Treg frequencies, and the frequency of CD86^+^ pDCs when adjusting for age, sex, HIV exposure, and for multiple comparisons (Supplementary Table 1; significance *P* < 0.017). However, CMV viral load correlated strongly with HLADR/CD38 coexpression on CD8^+^ T cells (coefficient = 15.05, *P* = 0.003) following adjustments age, sex, HIV exposure, and for multiple comparisons (Table 3 and Fig. 2e). No significant correlations were observed between CMV viral load and any other immunological outcomes (Table 3).

### Among HIV-exposed-uninfected infants, maternal health markers were not associated with detection of cytomegalovirus viraemia

To assess effects of maternal health on detection of CMV viraemia, we assessed maternal HIV-1 viral load at infant birth, ART duration, CD4^+^ T-cell count at infant birth and BMI at infant birth. Maternal data was only available for HEU infants. No significant differences were observed in maternal health indicators between groups based on the presence of infant CMV viraemia (Supplementary Table 3).

## Discussion

Much attention has been paid to the impact of HIV and ART exposure on adverse delivery outcomes [35,36], metabolic [37,38] and haematological [39,40] abnormalities, and immunological alterations [41,17] in HEU infants. The impact of maternal coinfections on HEU infant health is relatively underexplored and may directly impact some of the outcomes mentioned above. We assessed the burden and possible risk factors for systemic CMV replication in infancy in HEU and HUU infants and explored associations between CMV viraemia and anthropometric, haematological, and immunological outcomes. Our main findings were: a six-fold increased odds ratio of CMV viraemia in HEU infants compared to HUU infants; a strong negative association between CMV viral load and infant age; significant negative associations between CMV viral load and infant WAZ and head circumference-for-age Z-scores; and a significant positive association between CMV viral load and CD8^+^ T cell activation.

CMV viraemia has been detected in more than 90% of Kenyan [14] and Gambian infants (HEU and unknown HIV exposure, respectively) [42], and in lower proportions in South African [43] and Zambian [44] infants. A recent retrospective study of a large number of HEU and HUU infants from the Zimbabwe Vitamin A for Mothers and Babies Project (ZVITAMBO) cohort [45] found similar high CMV prevalence rates at 6 weeks-of-age, though no significant differences between well matched HEU and HUU infants. However, significantly higher median viral loads were observed in HEU infants. Our contrasting observations may be best explained by differences in background levels of CMV viraemia (74% of HUU infants at 6 weeks-of-age vs 45% of 2 to 8-month-old infants in our study), highlighting the importance of region-specific studies. In addition, the focus on one time point in the ZVITAMBO study may have led to underestimations of CMV viraemia in infant groups, limiting the ability to detect differences throughout infancy and assess the effect of age in modulating CMV viral load. Nevertheless, the study was conducted in an era before ART was available for PMTCT and in this setting, differences between HEU and HUU infant groups, such as median infant CMV viral load, may have been more pronounced.

We found infant age to be a critical factor associated with CMV viral load control, suggesting a role for de novo infant immunity in limiting systemic viral replication. However, extended low-level replication has been reported in longitudinal assessments of CMV viremia in infancy [14], suggesting functional limitations of de novo CMV-specific adaptive response, particularly in CD4^+^ T cells [46]. As CMV viral load is associated with clinical sequelae following congenital infection [47] and symptomatic acute infection [44], longitudinal studies are needed to evaluate the relationship between age of primary infection, peak CMV viral load, and clinical and immunological outcomes of HIV-exposed and unexposed infants.

We assessed the impact of viral load on anthropometry adjusting for age, sex, and HIV exposure status. In a previous study comparing HIV-exposed and unexposed Zambian infants, CMV infection was associated, at 18 months of age, with reduced HAZ scores irrespective of HIV exposure, and with reduced head circumference-for-age scores and psychomotor development in HEU infants [48]. Furthermore, the prevalence of stunting at 18 months of age was found to be three times higher in CMV seropositive HIV-exposed and unexposed children. Similarly, breast milk CMV load has been inversely correlated with HAZ and WAZ-scores at 6 months of age in HEU infants [49]. Accordingly, our data indicate a significant negative association between CMV viral load and WAZ and head circumference-for-age Z-scores. Our analysis limits causative inferences to be made, however, the consistency of the association of anthropometric parameters with CMV viral load across studies (reviewed in [50]) indicates a negative influence on infant growth from an early age. Studies assessing CMV viral load and neurocognitive outcomes in infancy are warranted.

Numerous studies describe phenotypic changes in ex-vivo leucocytes in HEU infants [51–53]. In particular, increased levels of activated (CD38^hi^) and antigen-experienced (CD45RO^+^) CD8^+^ T cells have been reported in HEU infants and adolescents [19,20], though not consistently [24,54,55]. As CMV infection is associated with broad phenotypic changes in CD8^+^ T cells [21,56], including activated and antigen-experienced cells, we speculated that CMV infection impacts early immune phenotypes in HEU and HUU infants. We observed a strong correlation between CD8^+^ T-cell activation and CMV viral load, indicating that CMV infection may influence bulk CD8 T-cell phenotypes nonspecifically. Coupled with an increased likelihood of early CMV viraemia in HEU infants, these data suggest that CMV infection may be a strong driver of cellular immune activation in HEU cohorts. Recent findings have also suggested that generalized inflammation, measured through C-reactive protein levels, is modulated by maternal HIV-1 viraemia more strongly than by infant CMV viraemia [45]. Future studies are needed to better understand the complex relationship between HIV exposure, immune activation/inflammation and, potentially, infant growth [57]. Our findings support the idea that CMV viraemia may serve an important correlate in these studies, and in future studies assessing infant T-cell immunity. Our data also support the notion that CMV viral load may be important when assessing infant outcomes in the context of infant malnutrition, as immune activation and inflammation are increased in malnourished infants [58].

Our data has several strengths and limitations. Strengths include the combination of anthropometric, detailed immunological and virological data in a cohort of HEU and unexposed control infants from the same ethnic group and community. Limitations include a cross-sectional evaluation over a narrow age, the consequent lack of information about the timing of CMV transmission, assessment of CMV viraemia in a single compartment and lack of clinical follow-up including data on prematurity. Based on the low prevalence of congenital CMV acquisition in other studies [42,49,59,60], we assume that in this study the majority of infections were primary postnatal infections, most likely through breastmilk exposure. In addition, consistent with an acute viral infection, we observed a strong positive effect of the presence of CMV viraemia on lymphocyte percentage. However, we cannot exclude the possibility of observing systemic viral replication because of CMV end-organ disease, possibly associated with preterm delivery, as the low HAZ scores reported may suggest prematurity or in-utero effects. Furthermore, we were unable to evaluate breastfeeding practices, a key factor associated with postnatal CMV acquisition. However, we did evaluate the influence of markers of maternal health on detection of CMV viremia, albeit only in HEU infants. Maternal immunosuppression and HIV-1 viremia has been associated with CMV shedding in cervical secretions, saliva, and breastmilk [61–63]. We found no association between maternal health and detection of infant CMV viral load in early life. This may be a result of the low number of infants in the analysis and the high infection rates in this setting, which further reduced the number of infants in the group with no CMV viremia.

### Conclusion

We find a six-fold increased odds of early CMV viraemia in HEU infants compared with their unexposed counterparts. Our data also indicate that increased CMV viraemia is associated with impaired growth, as well as with CD8^+^ T-cell activation. Our findings, together with those of others, suggest that increased early CMV transmission may be an important factor in the poor health of HEU infants. Further longitudinal assessments of the effects of CMV on growth, development, and immunomodulation in early life in HEU and HUU populations are warranted.

## Acknowledgements

We primarily thank the infants and mothers who participated in the study. We also thank the staff at the Comprehensive Care and Research Clinic namely, Anne Njogu, Margaret Lozi, Salma Said, Jefwa Kithunga, and Conny Kadenge. Finally, we thank Greg Fegan for guidance with statistical considerations. This manuscript was submitted for publication with the permission from the Director of the Kenya Medical Research Institute (KEMRI).

M.G-K., E.N., A.S.H., J.A.B., B.C.U., and S.L.R-J. conceived the study protocol and designed the experiments; M.G-K., E.N., I.N., F.G., D.O., and T.J.E. performed the experiments; A.S.H., N.J.H, and T.J.E., performed clinical procedures; M.G-K., E.N., A.S.H., J.A.B., B.C.U., and S.L.R-J. analysed the data; all authors helped write and/or review the manuscript.

The work was supported by the Wellcome Trust (089567/Z/09/Z to M.G-K. and 095068/Z/10/Z to E.N. and WT079082 to B.C.U.)

### Conflicts of interest

There are no conflicts of interest.

## Supplementary Material

Supplemental Digital Content

## Figures and Tables

**Table 1:** Characteristics of HIV-exposed uninfected and HIV-unexposed uninfected infants recruited from a rural coastal region in Kenya.

Characteristic				
Demography and CMV infection	HEU (*n* = 36)	HUU (*n* = 29)	*P* value	Total (*n* = 65)
Female (%)	17 (47.2)	16 (55.2)	0.53	33 (50.8)
Age in months, median (IQR)	4.2 (3.3, 4.9)	3.5 (2.8, 4.5)	0.07	3.9 (3.2, 4.9)

Anthropometry, median (IQR)	HEU (*n* = 31)	HUU (*n* = 22)		Total (*n* = 53)^a^
Weight for age Z-scores	−0.7 (−1.0, 0.2)	−0.4 (−1.2, 0.5)	0.63	−0.6 [−1.1, 0.4]
Height for age Z-scores	−1.5 (−2.7, −0.2)	−1.2 (−2.8, 1.6)	0.22	−1.3 (−2.7, 0.4)
Weight for height Z-scores	0.6 (−0.4, 1.3)	0.1 (−1.0, 2.1)	0.53	0.6 (−0.5, 1.7)
Head circumference Z-scores	0.3 (−0.8, 0.8)	0.4 (−0.5, 1.8)	0.19	0.3 (−0.6, 1.0)

Haematology, median (IQR)	HEU (*n* = 30)	HUU (*n* = 28)		Total (*n* = 58)^a^
Red blood cell count (10^6^/μl)	4.3 (4.0, 4.8)	4.3 (3.8, 4.8)	0.80	4.3 (3.9, 4.8)
White blood cell count (10^9^/L)	10.4 (7.3, 12.8)	9.2 (7.5, 10.9)	0.28	9.7 (7.3, 11.7)
Haemoglobin count (g/dl)	10.0 (10.0, 11.0)	9.9 [9.1, 10.8]	0.31	10.0 [9.5, 11.0]
Platelet count (10^9^/l)	426.0 (315.0, 516.0)	472.0 (400.0, 568.0)	0.22	439.0 (335.0, 542.0)
Absolute lymphocyte count (10^9^/l)	6.8 (5.0, 9.6)	6.1 (4.9, 7.6)	0.04	6.6 (5.0, 8.0)
Lymphocyte %	73 (65, 76)	69 (63, 73)	0.16	72 (63, 75)
Absolute neutrophil count (10^9^/l)	1.7 (1.1, 2.4)	1.5 (1.3, 2.0)	0.91	1.6 (1.2, 2.2)
Neutrophil %	17.0 (13.0, 23.0)	17.8 (15.3, 23.7)	0.57	17.7 (14.0, 23.7)

Immunology, median (IQR)	HEU (*n* = 17)	HUU (*n* = 10)		Total (*n* = 27)^b^
% CD38^+^ HLADR^+^ CD8^+^ T cells	37.8 (23.3, 51.2)	48.6 (14.2, 63.1)	0.32	40.2 (22.5, 54.6)
% CD38^+^ HLADR^+^ CD4^+^ T cells	3.2 (2.4, 5.7)	6.4 (4.8, 8.3)	0.03	4.2 (2.9, 7.2)
% PD1^+^ CD8^+^ T cells	22.7 (13.3, 27.5)	28.3 (16.2, 40.9)	0.16	22.7 (13.8, 29.4)
% PD1^+^ CD4^+^ T cells	8.7 (5.0, 12.9)	12.7 (9.0, 13.2)	0.12	9.7 (5.7, 13.1)
% CD25^hi^ FOXP3^+^ CD4 T cells	4.5 (3.2, 5.5)	5.1 (2.8, 6.1)	0.65	4.6 (3.1, 5.9)
% CD86^+^ pDC	33.0 (20.3, 37.6)	26 (17.8, 38.4)	0.80	27.2 (19.2, 37.6)

CMV, cytomegalovirus; HEU, HIV-exposed uninfected; HUU, HIV-unexposed uninfected.

^a^Infants with missing anthropometry (*N* = 12) and haematology (*N* = 7) data.

^b^2 to 5-month-old infants only for whom ex-vivo immunophenotypic data had been previously generated by fresh whole blood immunophenotyping. χ^2^ tests were used for comparisons of categorical variables. Unpaired *t* tests or Mann–Whitney tests were used to compare continuous variables, depending on the distribution of the data.

**Table 2:** Correlates of being cytomegalovirus viraemic in all infants (model 1) and of the magnitude of cytomegalovirus viraemia (model 2) among viraemic infants.

Independent variable				
Model 1 (*N* = 65)	Crude OR (95% CI)	*P* value	Adjusted OR (95% CI)	^a^*P* value
HIV exposed uninfected	5.10 (1.69, 15.37)	0.004	5.95 (1.83, 19.36)	0.003
Female	0.48 (0.17, 1.36)	0.168	0.37 (0.11, 1.24)	0.108
Age (months)	1.19 (0.84, 1.67)	0.324	1.06 (0.74, 1.53)	0.740

Model 2 (*N* = 42)	Crude linear regression coefficient (95% CI)	*P* value	Adjusted linear regression coefficient (95% CI)	^b^*P* value
HIV exposed uninfected	0.28 (−0.12, 0.69)	0.161	0.27 (−0.10, 0.64)	0.142
Female	0.48 (0.17, 1.36)	0.168	0.11 (−0.26, 0.47)	0.549
Age (months)	−0.17 (−0.27, −0.06)	0.004	−0.15 (−0.27, −0.04)	0.009

CI, confidence interval; OR, odds ratio.

^a^Adjusted *P* values for multivariable logistic regression model including age, sex, and HIV exposure.

^b^Adjusted *P* values from multivariable linear regression model including age, sex, and HIV exposure.

**Table 3:** Correlations between cytomegalovirus vireamia and anthropometry, haematology and immunological parameters among cytomegalovirus viraemic infants.

Characteristics	^a^Adjusted linear regression coefficient ([95% CI)	*P* value
Anthropometry (*N* = 33)^a^		
Weight for age Z-scores	−1.06 (−1.82, −0.29)	0.008
Height for age Z-scores	−2.36 (−4.52, −0.188)	0.034
Weight for height Z-scores	1.10 (−0.20, 2.41)	0.094
Head circumference Z-scores	−1.47 (−2.59, −0.35)	0.012
Haematology (*N* = 42)^b^		
Red blood cell count (10^6^/μl)	0.02 (−0.05, 1.01)	0.474
White blood cell count (10^9^/L)	0.22 (−0.01, 0.45)	0.058
Haemoglobin count (g/dl)	0.04 (−0.04, 0.11)	0.342
Platelet count (10^9^/l)	0.26 (−0.25, 0.77)	0.305
Absolute lymphocyte count (10^9^/l)	0.21 (−0.07, 0.50)	0.136
Lymphocyte %	0.89 (−7.38, 9.16)	0.826
Absolute neutrophil count (10^9^/l)	0.05 (−0.32, 0.42)	0.790
Neutrophil %	−2.01 (−9.89, 5.87)	0.605
Immunology (*N* = 19)		
% CD38^+^ HLADR^+^ CD8^+^ T cells	15.05 (6.10, 23.99)	0.003
% CD38^+^ HLADR^+^ CD4^+^ T cells	2.37 (0.34, 4.40)	0.026
% PD1^+^ CD8 T cells	6.33 (−3.49, 16.16)	0.189
% PD1^+^ CD4 T cells	1.07 (−3.33, 5.51)	0.606
% CD25^hi^ FOXP3^+^ CD4 T cells	−0.47 (−1.92, 0.98)	0.500
% CD86^+^ pDC	8.35 (−1.87, 18.58)	0.102

CI, confidence interval.

^a^Values are adjusted for HIV exposure, age, and sex.

^b^Infants with missing anthropometry (*n* = 12) and hematology (*n* = 7) data.
