# Supplementary material for: Cytomegalovirus viraemia is associated with poor growth and T-cell activation with an increased burden in HIV-exposed uninfected infants
Source: AIDS. 2017 Jul 27;31(13):1809–18. doi: 10.1097/QAD.0000000000001568 (PMC5538302; doi:10.1097/QAD.0000000000001568)
Supplement: Supplemental Digital Content [file aids-31-1809-s001.doc]

**Supplementary information**

**Cytomegalovirus viraemia is associated with poor growth and T-cell activation with an increased burden in HIV-exposed uninfected infants**

Miguel GARCIA-KNIGHT, Eunice NDUATI, Amin S. HASSAN, Irene NKUMAMA, Timothy J. ETYANG, Naseem J. HAJJ, Faith GAMBO, Denis ODERA, James A BERKLEY, Sarah L. ROWLAND-JONES, Britta URBAN


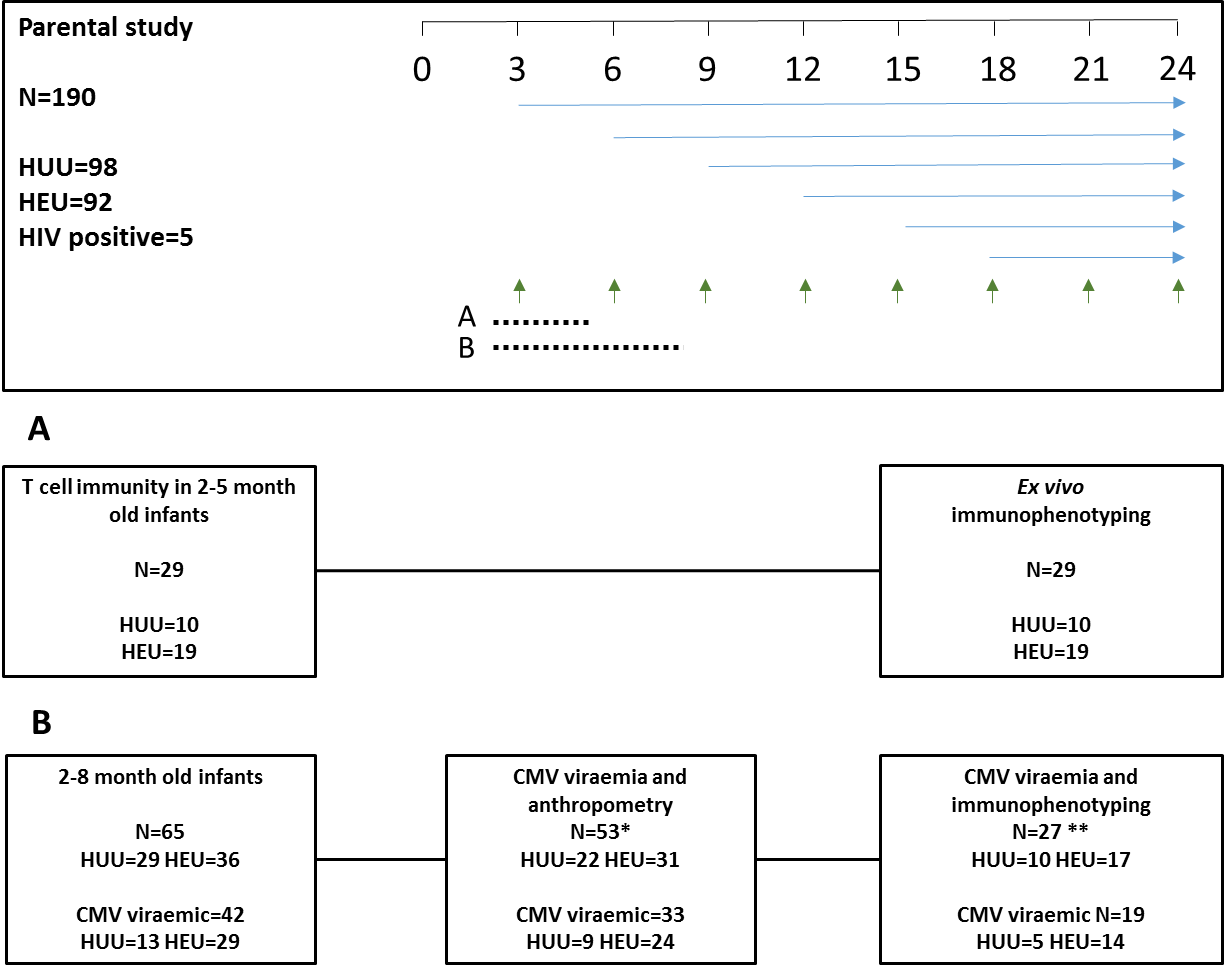


**Supplementary figure 1: Inclusion of infant specimens in the current study in relation to previous studies and the parental cohort.** A consecutive sampling approach was used in the parental cohort (upper panel) to recruit HIV-exposed uninfected (HEU) infants between 3 and 18 months of age and follow them until 24 months (blue arrows). HIV-unexposed uninfected (HUU) infants were recruited cross sectionally (green arrows). (A) A study on T cell immunity in 2-5-month-old infants (dotted line A in the upper panel) in which the *ex vivo* immune activation data was generated was previously reported (Garcia-Knight et al PLoS One 2015 [24]). (B) The present study included the recruitment sample of all infants between 2 and 8 months of age (dotted line B in the upper panel), who remained HIV-1 negative throughout follow-up and who had a cryopreserved plasma sample. *Anthropometry data not recorded for 12 infants. **No plasma sample was available for CMV diagnosis in two infants aged 2-5 months.

D)

**Supplementary figure 2. Gating strategy used to define populations of activated T cells and CD4+ T regs in peripheral whole blood.** (A) Lymphocytes were identified using forward and side scatter profiles and cell doublet events were excluded. (B) CD3+ CD4 and CD8 T cells were identified. CD3 Pacific blue, CD4 PerCP and CD8 APCH7 conjugates were used. C) Isotype control stains were used to set gates in order to define the frequency of CD4 and CD8 T cell populations co-expressing the activation markers CD38 and HLA-DR and the exhaustion marker PD-1 conjugated to the indicated fluorochromes. The staining panel also included the cell surface marker Tim3 PE (not shown). D) Using a separate staining panel, CD3+ CD4+ CD25hi cells were gated on and an isotype control was used to define FOXP3+ events on a CD3 vs FOXP3 dot plot.

B)

C)

Relative cell number

**Supplementary figure 3. Gating strategy used to determine the frequency and phenotype of pDCs** A) Whole blood leucocytes were identified and singlet populations of cell events included. Dendritic cells were defined as lineage marker negative (Lin), HLA-DR positive. B) pDC were further defined as CD123 positive using the fluorochrome efluor 450 (eF). C) CD86 positive events were defined through the use of isotype controls.

**Supplementary table 1. Correlation between being CMV viraemic and anthropometry and haematology parameters**

| **Characteristics** | **Adjusted linear regression coefficient [95% CI]*** | **P value** |
| --- | --- | --- |
| *Anthropometry (N=53)*** | | |
| Weight for age z-scores | 0.63 (-0.11, 1.36) | 0.09 |
| Height for age z-scores | 0.11 (-1.28, 1.50) | 0.87 |
| Weight for height z-scores | 0.65 (-0.54, 1.83) | 0.28 |
| Head circumference z-scores | -0.07 (-1.06, 0.92) | 0.89 |
| *Haematology (N=58)*** | | |
| Red blood cells count (10^6^/μL) | 0.05 (-0.01, 0.11) | 0.09 |
| White blood cells count (10^9^/L) | 0.05 (-0.12, 0.22) | 0.52 |
| Haemoglobin count (g/dL) | -0.00 (-0.06, 0.06) | 0.99 |
| Platelet count (10^9^/L) | 0.01 (-0.42, 0.44) | 0.972 |
| Absolute lymphocytes count (10^9^/L) | 0.14 (-0.07, 0.34) | 0.181 |
| Lymphocyte % | 12.08 (3.52, 20.65) | 0.007 |
| Absolute neutrophils count (10^9^/L) | -0.29 (-0.56, -0.02) | 0.033 |
| Neutrophils % | -5.75 (-11.54, 0.05) | 0.052 |
| *Immunology (N=27)* |  |  |
| % CD38+ HLADR+ CD8 T cells | 22.00 (3.60, 40.40) | 0.021 |
| % CD38+ HLADR+ CD4 T cells | -0.82 (-4.28, 2.64) | 0.628 |
| % PD1^+^ CD8 T cells | 12.68 (0.97, 24.39) | 0.035 |
| % PD1^+^ CD4 T cells | -1.40 (-6.72, 3.92) | 0.588 |
| % CD25^hi^ FOXP3^+^ CD4 T cells | -1.66 (-3.12, -0.21) | 0.027 |
| % CD86^+^ pDC | 12.44 (1.65, 23.22) | 0.026 |

*Values are adjusted for HIV exposure, age and sex **Infants with missing anthropometry (n=12) and hematology (n=7) data;

**Supplementary table 2**: Comparison between infants included in the T-cell immune activation marker sub-study and, those not included in the sub study and the overall cohort

| **Characteristics** | | **T-cell sub-study**  **(No, n=38)** | **T-cell sub-study**  **(Yes, n=27)** | **Total**  **(n=65)** | **p value*** |
| --- | --- | --- | --- | --- | --- |
| *a)Demography (N=65)<* | | | | |  |
| HIV exposure | HUU  HEU | 19 [50.0]  19 [50.0] | 10 [37.0]  17 [63.0] | 29 [44.6]  36 [55.4] | NS |
| Gender (%) | Female  Male | 18 [47.4]  20 [52.6] | 14 [51.9]  13 [48.2] | 32 [49.2]  33 [50.8] | NS |
| Age (in months) | Median [IQR] | 4.5 [3.4, 6.8] | 3.5 [3.0, 4.1] | 3.9 [3.2, 4.9] | <0.01 |
| *b) Anthropometry, median [IQR] (N=53)** | | | | | |
| Weight for age z-scores | | -0.5 [-1.1, 0.4] | -0.7 [-1.0, 0.2] | -0.6 [-1.1, 0.4] | NS |
| Height for age z-scores | | -1.0 [-2.9, 1.0] | -1.5 [-2.1, 0.8] | -1.3 [-2.7, 0.4] | NS |
| Weight for height z-scores | | 0.2 [-0.7, 1.7] | 0.6 [-0.5, 1.7] | 0.6 [-0.5, 1.7] | NS |
| Head circumference z-scores | | 0.2 [-0.8, 1.0] | 0.3 [-0.4, 1.0] | 0.3 [-0.6, 1.0] | NS |
| *b) Hematology, median [IQR] (N=58)** | | | | | |
| Red blood cells count (10^6^/μL) | | 4.3 [4.0, 4.8] | 4.3 [3.8, 4.7] | 4.3 [3.9, 4.8] | NS |
| White blood cells count (10^9^/L) | | 10.4 [8.0, 11.7] | 8.6 [6.5, 11.0] | 9.7 [7.3, 11.7] | NS |
| Hemoglobin count (g/dL) | | 10.0 [9.5, 11.0] | 10.1 [9.5, 11.0] | 10.0 [9.5, 11.0] | NS |
| Platelets count (10^9^/L) | | 427 [324, 506] | 493 [363, 575] | 439 [335, 542] | NS |
| Absolute lymphocytes count (10^9^/L) | | 6.5 [5.4, 7.8] | 6.6 [4.4, 8.8] | 6.6 [5.0, 8.0] | NS |
| Percent lymphocyte count | | 69.5 [62.0, 75.0] | 73.0 [69.0, 75.0] | 71.7[63.0, 75.0] | NS |
| Absolute neutrophils count (10^9^/L) | | 1.8 [1.3, 2.7] | 1.4 [1.2, 1.9] | 1.6 [1.2, 2.2] | NS |
| Percent neutrophils count | | 19.5 [13.6, 25.7] | 17.0 [14.7, 21.0] | 17.7 [14.0, 23.7] | NS |
| Absolute monocytes count (10^9^/L) | | 0.7 [ 0.5, 0.9] | 0.6 [0.4, 0.7] | 0.7 [0.5, 0.8] | NS |
| Percent monocyte count | | 6.2 [5.0, 7.4] | 6.0 [5.0, 7.5] | 6.1 [5.0, 7.5] | NS |
| Eosinophil count (10^9^/L) | | 0.3 [0.2, 0.6] | 0.2 [0.2, 0.3] | 0.3 [0.2, 0.5] | NS |

NS, not significant

Chi squared tests were used for comparisons of categorical variables. Unpaired t tests or Mann-Whitney tests were used to compare continuous variables, depending on the distribution of the data

*Comparison between T cell study and total infant population

**Infants with missing anthropometry (HEU [n=5] and HUU [n=7]) and haematology (HEU [n=6] and [HUU n=1]) data.

**Supplementary table 3. Influence of maternal characteristics on CMV** viremia in HEU infants

| **Characteristic** | **Not CMV viraemic (n=7)** | **CMV viraemic (n=22)** | **P value** |
| --- | --- | --- | --- |
| Median log10 HIV RNA copies/µL (IQR) | 2.9 (0, 4.5) | 3.0 (0, 3.9) | 0.8 |
| Duration of maternal ART in months (IQR) | 18.0 (11.5, 29.8) | 28.0 (7.4, 55.0) | 0.7 |
| Maternal CD4 count at infant birth (cells/µL) | 300.5 (213.0, 674.0) | 421.0 (300.0, 690.0) | 0.4 |
| Maternal BMI at infant birth | 21.0 (20.5, 27.2) | 21.5 (20.2, 23.2) | 0.6 |

Unpaired t tests or Mann-Whitney tests were used to compare continuous variables, depending on the distribution of the data.
